# Characteristics of Cracking Failure in Microbump Joints for 3D Chip-on-Chip Interconnections under Drop Impact

**DOI:** 10.3390/mi13020281

**Published:** 2022-02-10

**Authors:** Zhen Liu, Mingang Fang, Lei Shi, Yu Gu, Zhuo Chen, Whenhui Zhu

**Affiliations:** 1 The School of Mechanical and Electrical Engineering, Central South University, Changsha 410083, China; zhenliu@csu.edu.cn (Z.L.); mingangfang@csu.edu.cn (M.F.); 2 Hunan Vocational College of Science and Technology, Changsha 410004, China; 3 China Electronic Technology Corporation (CETC) 48 Research Institute, Changsha 410083, China; iheartlan23@163.com; 4 Qingdao Electronics School, Qingdao 266014, China; guyu_qddz@aliyun.com

**Keywords:** crack propagation, microbump, deflection angle, stress intensity factor (SIF)

## Abstract

With the rapid development of microelectronics packaging and integration, the failure risk of micro-solder joints in packaging structure caused by impact load has been increasingly concerning. However, the failure mechanism and reliability performance of a Cu-pillar-based microbump joint can use little of the existing research on board-level solder joints as reference, due to the downscaling and joint structure evolution. In this study, to investigate the cracking behavior of microbump joints targeted at chip-on-chip (CoC) stacked interconnections, the CoC test samples were subjected to repeated drop tests to reveal the crack morphology. It was found that the crack causing the microbump failure first initiated at the interface between the intermetallic compound (IMC) layer and the solder, propagated along the interface for a certain length, and then deflected into the solder matrix. To further explore the crack propagation mechanism, stress intensity factor (SIF) of the crack tip at the interface between IMC and solder was calculated by contour integral method, and the effects of solder thickness and crack length were also quantitatively analyzed and combined with the crack deflection criterion. By combining the SIF with the fracture toughness of the solder–Ni interface and the solder matrix, a criterion for crack deflecting from the original propagating path was established, which can be used for prediction of critical crack length and deflection angle for the initiation of crack deflection. Finally, the relationship between solder thickness and critical deflection length and deflection angle of main crack was verified by a board level drop test, and the influence of grain structure in solder matrix on actual failure lifetime was briefly discussed.

## 1. Introduction

Three-dimensional (3D) integration of silicon dies or wafers has received considerable attention in the past decade, due to its advantages of higher I/O density, lower RC delay, capability of heterogeneous integration, and footprint shrinking. Microbumps containing solder alloys have been deployed for establishing electrical and mechanical connection between vertically stacked chips [1,2]. Although similar in principle to the well-developed flip-chip technology, the interconnections using microbumps are still subjected to process adaptations. Therefore, solder joint reliability plays a vital role in the quality of electronic products.

Among all reliability issues, drop impact reliability of a solder joint, in particular, is of great importance and has attracted many researchers. For ball grid array (BGA) level solder joints typically 200–500 μm in size, the main failure mode during drop impact loading is manifested as cracking along the interface of solder bump and the intermetallic compounds (IMCs) formed by soldering [3,4], and the joint at the outermost corner is found as the most critical, which fails along the solder–pad interface [5,6]. F. X. Che et al. found that the constitutive model of solder used in the input-G simulation has a major impact on the stress and strain in a solder joint and on the hardening effect of bulk solder under a high strain rate during drop impact, which prevents the drop impact energy from dissipating through the bulk solder and accounts for the interface cracking [7]. However, downsizing of the interconnection joint size entails the reconsideration of a failure mechanism and characteristics of the micro-interconnections, as the joints in a chip-on-chip stacking scenario could use little of the previous studies at a larger scale as direct reference. Therefore, recent research of drop reliability also focuses on the 3D die-stacking structure. This includes the study by Chen et al. who determined that the critical position under the board-level drop impact is the corner of bottom layer of copper via [8], and the reliability improvement with a thinner IMC layer was revealed by Hsien-Chie Cheng et al. [9,10]. They also found that the interconnects under the drop test would exhibit a cohesive fracture inside the solder, which is different from the BGA cases studied by Suh [6,11]. M. O. Alam studied the parameters of stress intensity factors (SIF, *K*_I_ and *K*_II_) around predefined cracks in the IMC layer of a solder butt joint by using linear elastic fracture mechanics (LEFM) and found that the SIF values increase sharply when the placement of the crack approaches near the interface. In summary, the reliability of microbumps for 3D integration under drop impact draws increasing concerns in interfacial fracture mechanics, as the cracking is strongly affected by the interfacial mechanical mismatch, and the propagation path will be complexly determined by both the interface feature and the solder matrix. Some research works have involved the path selection of the crack near the interface [12], but there is no description of the dynamic process of crack propagation near the interface.

In this study, we first observed the crack failure of a microbump joint in a chip-on-chip (CoC) test vehicle under drop test conditions and found that the crack formed at the edge of the soldering interface, propagated along the interface for a certain length, and deflected into the solder layer, eventually causing failure of the joint. To elucidate this phenomenon, a finite-element model was constructed to investigate the crack propagation behavior, based on basic fracture mechanics theories. The stress intensity factor of the crack tip at the interface between the IMC and solder is calculated by the contour integral method, and the propagation path of the solder joint interface crack is studied by using the criterion of energy release rate versus the fracture toughness in both the original and the deflected propagation path. Experimental tests for the joints of different solder thicknesses were carried out and compared with the numerical calculations to validate the model. Finally, the experimental observations revealed how the grain structure of the solder layer may affect the actual cracking path and drop lifetime.

## 2. Setup for Drop Experiment

**Figure 1:**
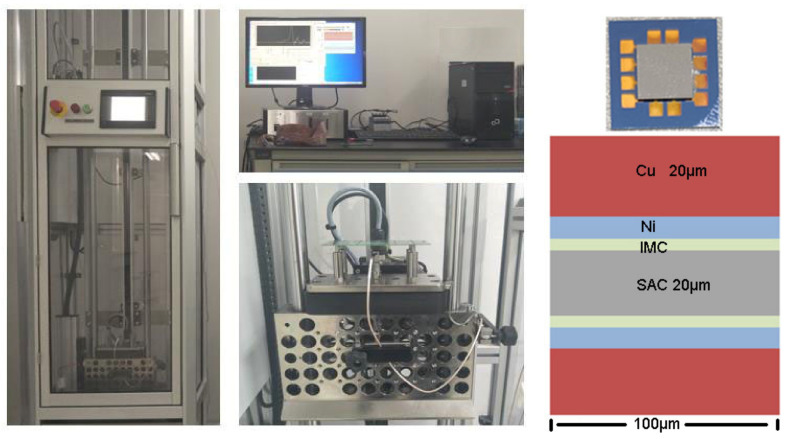
The schematic diagram of drop test and the cross-section images of the unit of the daisy chain.

Figure 1 shows the schematic diagram of the drop experiment. A Chip-on-chip test vehicle was used, which consisted of a 6 × 6 × 0.5 mm top chip, and a 12 × 12 × 0.5 mm bottom chip. Both the top chip and bottom chip had a microbump array fabricated on the surface through a standard lithography–etching–electroplating bumping process. Each microbump consisted of a Cu pillar, a Ni barrier layer, and a SnAg cap. The two chips were bonded through a flip-chip thermo compression process by an Athlete CB-600 flip-chip bonder with an alignment accuracy of ±1 μm. The temperatures of the bonding head and bottom suction tool were set at 340 and 100 °C to obtain a peak temperature of 260 °C at the soldering interface, and the bonding pressure was 0.06 N per bump. Target temperature and pressure were applied for 30 s. The 5 μm Cu traces on both chips linked each bump to form two daisy chains, each comprising 24 pair of bumps.

**Figure 2:**
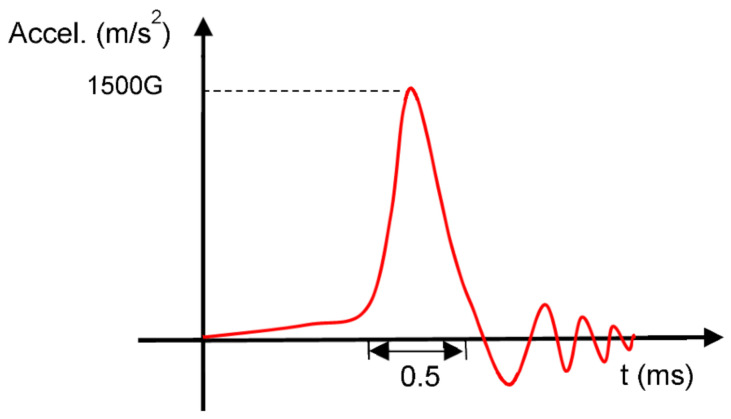
Impact acceleration of test results.

A JEDEC-compliant Salon Teknopajia drop tester executed the drop experiments. The CoC module was firmly assembled on the center of test board where the impact-induced distortion is highest. The dimension of the test board also complies with the JEDEC standard, although only the 1-chip arrangement was used. A daisy chain in the module was electrically connected to a high speed data acquisition circuit to allow for transient resistance recording in real-time during drop test. The test board was then fastened onto the base plate by four screws. For each drop, the base plate was raised to the height specified in JEDEC standard and dropped on the strike surface with the acceleration G measured to follow the curve shown in Figure 2. For the observation of microstructure evolution, CoC modules after certain numbers of drops were cross-sectioned and examined under a field-emission scanning electron microscope (FE-SEM) working at the backscattered electron imaging mode.

## 3. Set up for Simulation and Experiment

**Figure 3:**
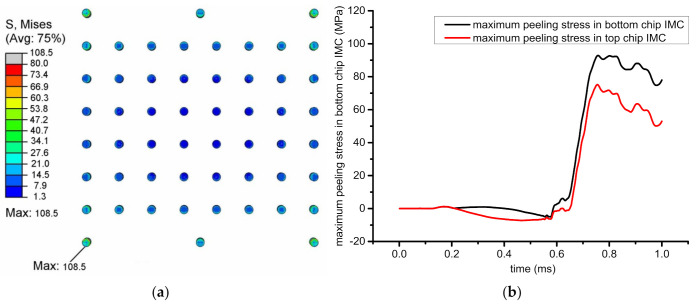
(**a**) Von Mises stress distribution of solder joint; (**b**) maximum peeling stress curves in a microbump joint during the impact load.

A finite-element (FE) code that employs transient dynamics was applied to investigate the mechanical response of the bump joint structure in a mechanical simulation for the drop test related above. The material properties in the model are all linear elastic models, as shown in Table 1. Von Mises stress distribution in the whole model at the moment of highest impact acceleration is shown in Figure 3a. According to the literature, in the board-level drop test of BGA, the failure of solder balls was mainly due to peel stress [13]. Here, the reliability of the microbumps is likewise focused by simulating the stress built in the joints between top and bottom chips. For the outer corner joints, which were subjected to highest impact stress, the maximum peeling stress is shown in Figure 3b. In the top-side, IMC was 75.8 MPa, while in the bottom, IMC of the same joints was 91.4 MPa. Therefore, the applied load was set from 10 to 90 MPa in the following FE model for the analysis of interfacial cracking behavior.

Because the solder joint has a cylindrical symmetry, the model for the calculation of the stress intensity factor at the crack tip is a two-dimensional model based on plane strain (Figure 1), which has an Sn-3.0Ag-0.5Cu solder(SAC305)–IMC–Ni sandwich configuration with dimensions of 100 × 20 μm, 100 × 1 μm, and 100 × 2 μm, respectively. A zero-thickness crack is preset at the interface between the IMC and solder layers, and the crack length is variable. The method of presetting the zero-thickness crack is the common point method. The surface morphology of IMC is ignored, and the interface between IMC and the solder is assumed to be flat. With an IMC thickness of only 1 μm, the possible void formation around the IMC layer was ignored, and the Ni–IMC interface was considered as ideal. The bottom of the copper pad is a fixed end, and a static-type tensile load is uniformly applied on the upper surface of the solder.

**Figure 4:**
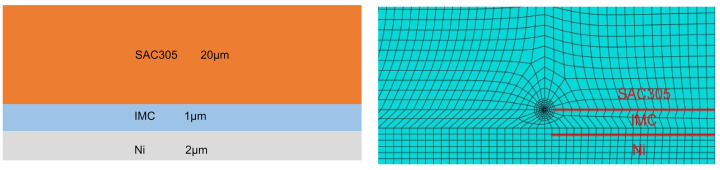
Model for calculating the stress intensity factor under different crack lengths.

The interaction integral method is used to solve the stress intensity factor at the crack tip. Because the crack in the model is on the interface between the IMC and the solder, the elasticity of the material on each side is different; thus, discontinuity appears on the interface. To ensure the calculation accuracy, the integral path of the contour is processed in sections. The mesh of the model adopts the region division method, and the smaller mesh size is used at the crack tip to ensure the solution accuracy, as shown in Figure 4. Affected by the thickness of the IMC, the mesh quality of the grid in the crack tip decreases sharply from the first to the fourth layer. Therefore, the average stress intensity factor calculated by taking the four integral contours at the innermost layer in the crack tip is utilized as the stress intensity factor around the crack tip.

## 4. Results and Discussion

### 4.1. Failure Mode and Mechanism of Microbumps

**Figure 5:**
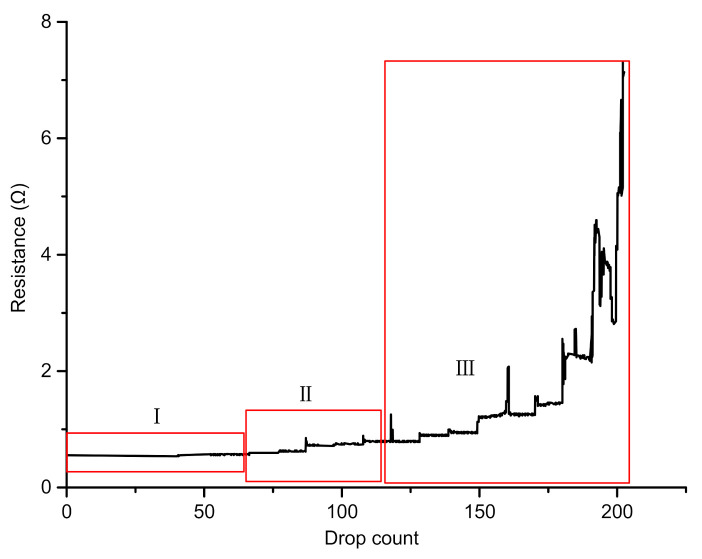
Typical resistance curves of daisy chains under drop test.

**Figure 6:**
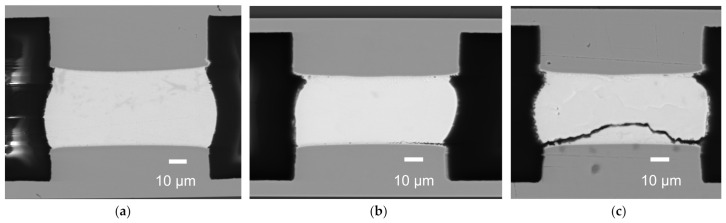
Cross-sectional SEM of the microbumps at different stages of drop impact. (**a**) Cross-sectional SEM of the sample without drop test; (**b**) after 50 drops; (**c**) after the circuit is completely disconnected.

In order to determine the failure characteristics of microbump interconnections under drop impact, first, the recording of the transient resistance of the daisy chain was plotted against the drop counts, as shown in Figure 5. The resistance change contains three distinct stages. Stage I denotes the period in which resistance value *R* remained unchanged; this stage typically lasts for the first 60 drops. Then, in several tens of following drops, denoted as stage II, fluctuation of R is detected, with the peak value not exceeding 120% of the original value. Later, R experiences a period of drastic fluctuation that it increases to far more than the initial value, and the daisy chain becomes completely open in less than 80 drop counts. In order to further explore the crack propagation mechanism, the drop samples were sliced and analyzed at different stages of circuit damage during continuous drop test. Figure 6a is a cross-sectional SEM of the sample without a drop test, and it can be seen from the figure that the IMC interface formed under the hot pressing bonding conditions used in the experiment is of good quality. As shown in Figure 6b,c, after the first 50 drops, a micro-crack was visible at the end of the IMC–solder interface of the bottom chip side. After the circuit was completely disconnected, a through crack could be observed. It can be concluded that the solder joint accelerated failure after crack propagation and deflection. Therefore, the resistance change pattern can be used to estimate the extent to which the structural damage of a critical microbump has progressed. It can also be seen that the joint degradation accelerated after the crack deflection since a significant spurt of resistance corresponds to the rapid shrinking of the residual joint area in this stage.

### 4.2. Stress Intensity Factor Analysis of Solder–IMC Interface Crack under Quasi-Static Load

#### 4.2.1. Relationship between Stress Intensity Factor at Interface Crack Tip and Crack Length

**Figure 7:**
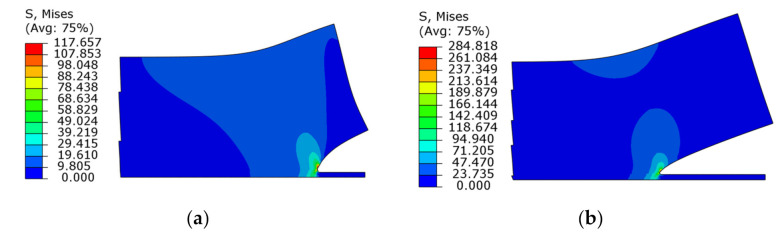
The Von Mises equivalent stress distribution in the crack tip with the crack length of: (**a**) 10 μm; (**b**) 20 μm.

Figure 7 shows the von Mises equivalent stress distribution at the crack tip when the load is 10 MPa and the crack length is 10 and 20 μm. It can be seen that the equivalent stress of the crack tip increases as the crack length increases, and the high equivalent stress appears both on the solder and IMC. However, it does not mean that failure or crack propagation will definitely occur in these locations. Under tensile load, the crack between the upper layer and the substrate initiate from the free edge of the actual specimen, especially where defects such as cracking or void brought by the bonding process existed. The initial crack first expands along the interface to a certain depth and then propagates along the interface or is deflected to the solder matrix, which depends on the energy release rate of the two propagation paths. Therefore, the energy release rate will be used to judge whether the crack is initiated and propagated, and the stress intensity factor will be used to determine the crack tip propagation path.

**Figure 8:**
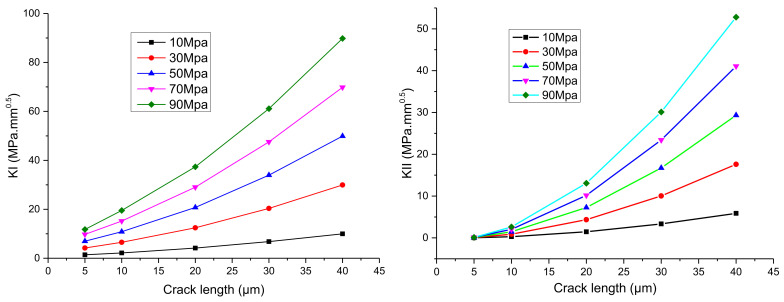
The relationship between the stress intensity factor of the interface crack between the IMC and the solder and crack depth.

The relationship between the stress intensity factor at the crack tip of the IMC–solder interface calculated by the interaction integral and the crack depth is shown in Figure 8. It can be seen that the stress intensity factors of *K*_I_ and *K*_II_ of the interfacial crack tip increase with an increase in the crack length under the same load, and the *K*_II_ will increase quickly due to the elastic deformation of the solder, which leads to the increasing tendency of the type II cracking mode and possibly the crack deflection as well.

Polynomial fitting is performed for the stress intensity factor at the crack tip with different crack lengths in the figure, and the fitting expression is as follows:

(1) $$K_{Ⅰ}=\left\{\begin{array}{ll} 3.7\times 10^{3}a^{2}+1.7\times 10^{2}a+0.013 & \sigma =10\mathrm{MP}a\\ 18.6\times 10^{3}a^{2}+8.5\times 10^{2}a+0.65 & \sigma =50\mathrm{MP}a\\ 33.3\times 10^{3}a^{2}+15.4\times 10^{2}a+1.2 & \sigma =90\mathrm{MP}a \end{array}\right.$$

(2) $$K_{Ⅱ}=\left\{\begin{array}{ll} 5.44\times 10^{3}a^{2}-1.27a-0.054 & \sigma =10\mathrm{MP}a\\ 27.2\times 10^{3}a^{2}-5.68a-0.265 & \sigma =50\mathrm{MP}a\\ 48.9\times 10^{3}a^{2}-9.77a-0.48 & \sigma =90\mathrm{MP}a \end{array}\right.$$

where σ is the peel stress loaded on the upper surface of the solder and a is the crack length. Comparing the stress intensity factors of *K*_I_ and *K*_Ⅱ_ under three loads, it can be seen that *K*_I_ and *K*_Ⅱ_ are proportional to the load, because the material model used in the simulation is a linear elastic model. Therefore, the expressions of *K*_I_ and *K*_Ⅱ_ can be rewritten as follows:

(3) $$\begin{array}{l} K_{Ⅰ}=3.7\times 10^{2}a^{2}\sigma +17a\sigma +0.013\sigma \\ K_{Ⅱ}=5.44\times 10^{2}a^{2}\sigma -0.127a\sigma -0.0054\sigma \end{array}$$

#### 4.2.2. Influence of Solder Thickness on Stress Intensity Factor of Interface Crack

**Figure 9:**
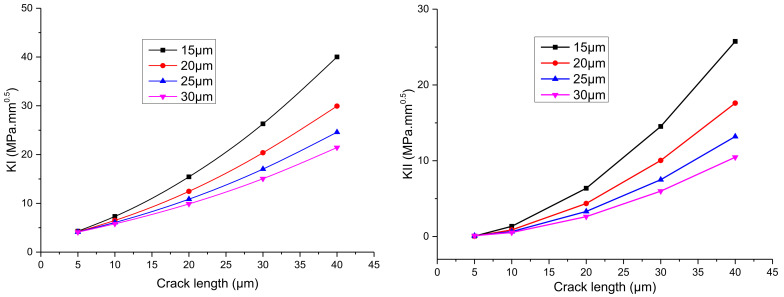
The relationship between the stress intensity factor *K*_I_ of the interfacial crack tip and solder thickness under different solder thicknesses.

**Figure 10:**
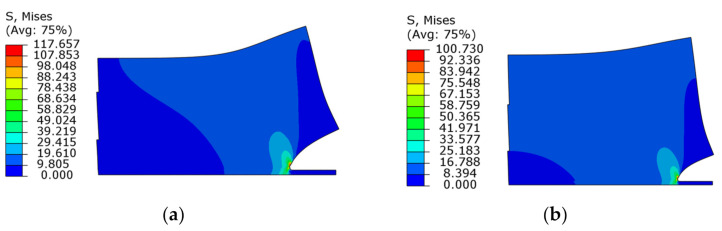
The von Mises equivalent stress distribution in the crack tip with different solder thicknesses: (**a**) 20 μm; (**b**) 30 μm.

In the existing research on solder joints of several hundred micrometers, due to the much lower elastic modulus of solder versus the rest part of a joint, the solder volume plays an important role in the mechanical properties of the microbumps. If the thickness of the solder layer is too small, the mechanical properties of the microbumps will be adversely affected. The solder thickness in a microbump-based die stacking 3D integration structure is greatly reduced compared to the flip chip interconnection, which necessitates the research on the dependence of SIF on the solder thickness quantitatively. Figure 9 compares the SIF evolution with a progressing crack under different solder thicknesses from 15 to 30 μm. It can be seen that both *K*_I_ and *K*_II_ increase with a decrease in solder thickness. This phenomenon is plainly explained by the stress distribution around the crack tip, as shown in Figure 10. The elastic mismatch between IMC and the solder causes stress concentration around the crack tip, which is better alleviated with a thicker solder layer, as can be judged from the more uniform distribution of stress across the cross section of analysis. Therefore, switching from the spherical solder bumps to the Cu pillar-based microbump joints is believed to pose additional failure risk under the drop impact condition.

### 4.3. Investigation on Crack Growth Behavior

The analyses above have revealed the increase in the stress intensity factors *K*_I_ and *K*_II_ with increasing crack length. Further investigation of the crack propagation behavior, especially the propagation path, needs the quantitative analyses on the crack tip energy release rates *J*_1_ and *J*_2_. Hu [15] found the propagation behavior of a semi-infinite plane crack at the interface of a two-phase material in 1989 and revealed that theoretically the crack deviated from the original main crack propagation path by a minimum length. They further deduced the relationship between the stress intensity factor after crack deflection and along the original path. The maximum energy release rate can be used to determine the crack deflection angle. The criterion of deflection of quasi-static interface crack propagation behavior is as follows:

(4) $$\frac{G_{S}}{G}>\frac{\Gamma }{\Gamma_{i}}$$

Among them: $G_{s}=J=\sqrt{J_{1}^{2}+J_{2}^{2}}$, $G=\frac{K_{Ⅰ}{}^{2}}{E^{*}}$, Γ is the fracture toughness of the solder, and $\Gamma_{i}$ is the fracture toughness of Ni_3_Sn_4_ IMC. In this paper, the maximum fracture toughness of solder SAC305 is set to be 295 N/m, which is measured by Loo [16]. To be able to directly compare the fracture toughness values of the Ni-Sn–IMC interface from the various existing research, the fracture toughness is converted into a critical stress intensity factor. For the Ni_3_Sn_4_ layer, a critical stress intensity factor of 4.22 ± 0.45 MPa m^1/2^ measured by Ghosh [14] was adopted, which equals 165.5 N/m; thus, we obtain $\frac{\Gamma }{\Gamma_{i}}=1.78$. It can be seen from the expression of the crack tip energy release rate that when the material is of linear elastic property, the ratio $\frac{G_{S}}{G}$ is irrelevant to load. For the convenience of calculation, the ERR is calculated with the uniaxial load of 50 MPa, and the IMC and solder thicknesses are set as 1 and 20 μm, respectively. The energy release rate at the interface crack tip under different crack lengths is calculated as follows:

For the homogeneous two-material interface:

(5) $$J_{1}=\frac{K\bar{K}}{E^{*}\cosh^{2}(\pi \varepsilon )}$$

(6) J2=−Re[Kriε]Im[Kriε]πεcosh2(πε)×[1−ν14μ1(1−e−2πε)+1−ν24μ2(e2πε−1)]

where ε is the oscillatory index

(7) $$\varepsilon =\frac{1}{2\pi }\ln(\frac{1-\beta }{1+\beta })$$

β is the second Dundurs’ constant

(8) $$\beta =\frac{\mu_{1}(\kappa_{2}-1)-\mu_{2}(\kappa_{1}-1)}{\mu_{1}(\kappa_{2}+1)+\mu_{2}(\kappa_{1}+1)}$$

and *κ* is Kolosov’s constant

(9) $$\kappa =\left\{\begin{array}{l} \frac{3-\nu_{p}}{1+\nu_{p}}\;\;\mathrm{plane}\;\mathrm{stress}\\ 3-4\nu_{p}\;\mathrm{plane}\;\mathrm{strain} \end{array}\right.$$

where

(10) $$\frac{1}{E^{*}}=[\frac{1-\nu_{1}}{4\mu_{1}}+\frac{1-\nu_{2}}{4\mu_{2}}]$$

**Figure 11:**
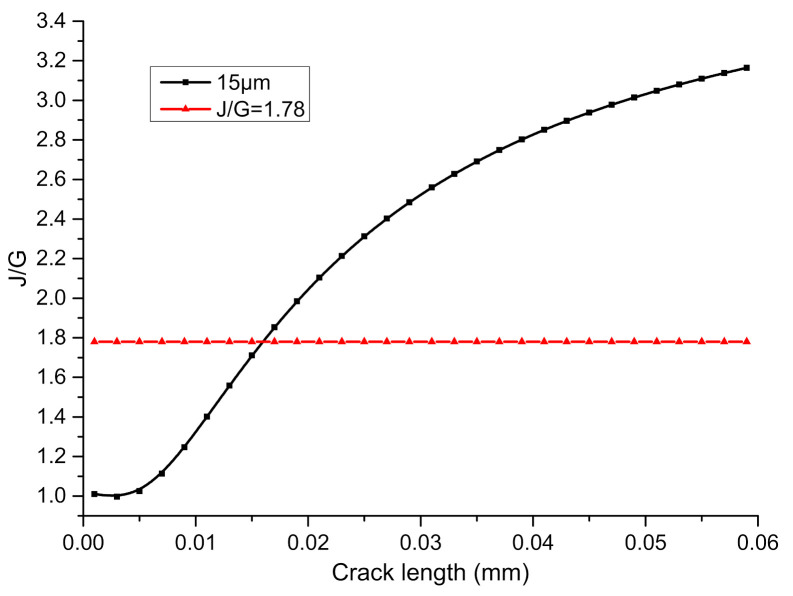
The variation trend of $\frac{G_{S}}{G}$ with crack length.

The results of the relevant parameters of the dual-material SAC305–IMC interface in the above formula are shown in Table 2. The trend of $\frac{G_{S}}{G}$ with crack length is calculated, as shown in Figure 11.

Hu found that the interface cracks start from the free edge of the sample, propagate at one to two times the thickness of the film along the interface, and then deflect into the matrix, expanding to a depth of four to five times the thickness of the film and finally parallel to the interface. From Figure 11, it can be seen that the ratio of the crack tip ERR after deflection to that propagating along the interface increases with the increase in the main crack length. When the main crack expands to a length of about 16 μm, the ratio will be greater than the ratio of the fracture toughness of the solder matrix to the fracture toughness of the interface. At this time, the crack will deviate from the original interface path and deflect into the matrix. The deflection angle is calculated by $\omega =\arctan\left|\frac{J_{2}}{J_{1}}\right|$, and we can find *w* = 42°. It can also be seen from the above figure that if the ratio of the fracture toughness of the solder matrix to the interface fracture toughness is greater than the ratio between two paths, then the crack will always expand along the interface without deflecting to the solder matrix.

**Figure 12:**
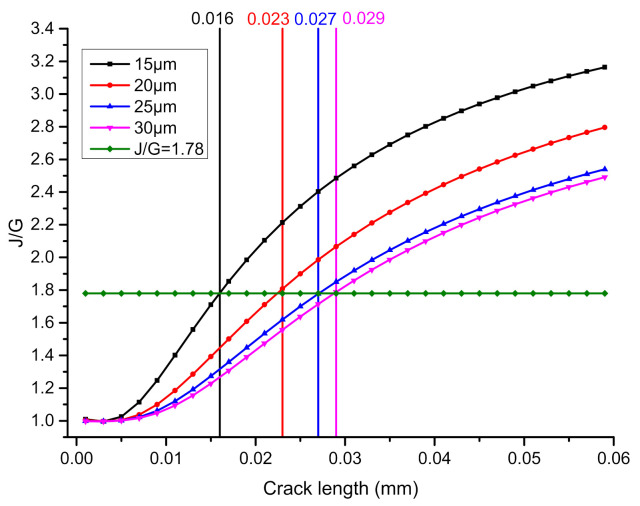
The influence of different solder thicknesses on interfacial crack growth behavior.

Figure 12 compares the influence of solder thickness on the interfacial crack growth behavior. It can be seen from the figure that when the solder thickness decreases, the critical main crack length for crack deflection will decrease. When the solder thickness is 15, 20, 25 and 30 um, the critical crack deflection length is 16, 23, 27 and 29 um, respectively, due to the reason related in Section 4.2.2, i.e., the decrease in solder cushioning causes an increase in stress concentration in the solder matrix, thus increasing the advantage of deflected cracking path.

**Figure 13:**
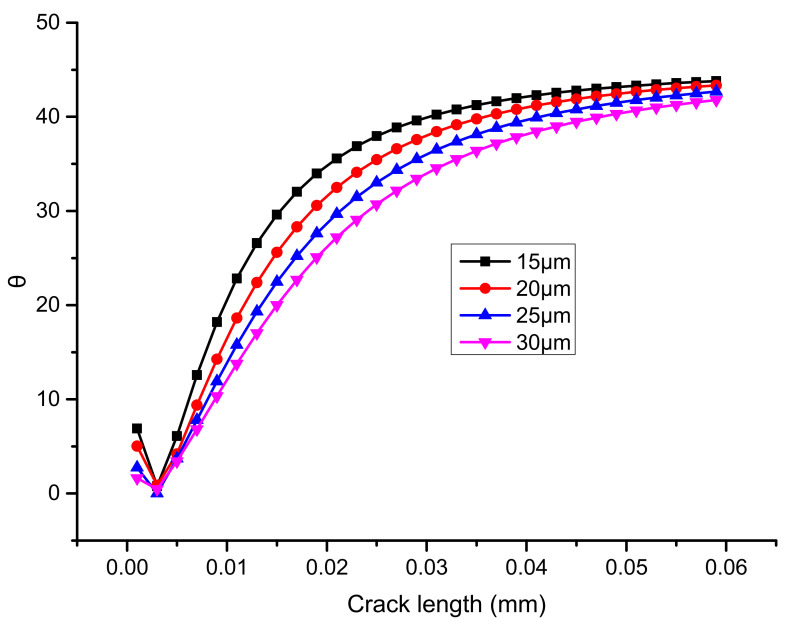
Relationship between the crack deflection angle and the main crack length under different solder thicknesses.

Figure 13 shows the variation of arctan |J2/J1|, or in other words, the virtual crack deflection angle, whether or not deflection actually takes place. With the main crack length under different solder thickness conditions, the angle increases rapidly at first, and then closes to a constant value. The crack deflection angle trend is consistent with the research of HH YU et al. on the interfacial cracking behavior of chromium films on silica substrates [12]. The asymptotic value of the crack deflection angle is about 42°.

## 5. Experimental Validation and Discussion

**Figure 14:**
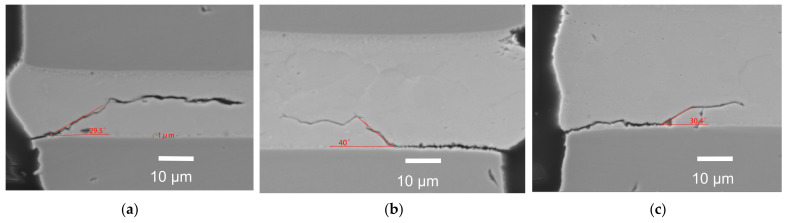
Cross section of the solder joint with different solder thicknesses, listed as: (**a**) 20 μm; (**b**) 30 μm; (**c**) 37 μm.

The cross section of the solder joint in the case of drop failure with different solder thickness is shown in Figure 14. According to the SEM analysis, when the solder thickness is 20 μm, the crack length of 8 μm deflects, and the deflection angle is 32.8°. When the solder thickness is 30 μm, the crack length of 28 μm deflects, and the deflection angle is 37°. When the solder height is 37 μm, the crack length of 32 μm deflects, and the deflection angle is 30.4°. The measured deflection angle of the interfacial crack is 30° to 40°, which is larger than the asymptotic value of deflection angle at the moment of deflection initiation, calculated by numerical simulation. This is owed to the microbump not only being subjected to normal stress, but it is also subjected to a shear force parallel to the interface during the drop experiment, while the load used in the numerical calculation is only the normal stress. In practice, when the crack propagates to a certain length, the portion of type II cracking produced by the shear stress cannot be negligible. The change of initial deflection angle versus the solder thickness is in good agreement with the numerical calculation based on ERR and fracture toughness. Therefore, in general, the numerical methods adopted in this paper can be used as an effective way to predict the cracking behavior in an actual microbump joint.

**Figure 15:**
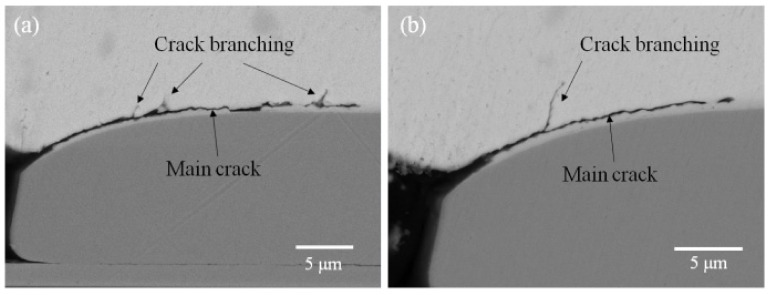
Crack branching during the initial stage of the drop test: (**a**) The minute crack branching increase as the fracture progresses; (**b**) The first crack branching.

The actual crack propagation behavior is affected by many factors, such as interfacial defects or inhomogeneity of microstructure. There is a clear competition between interfacial propagation and solder matrix propagation; for example, it was found in the test vehicles of inferior interfacial strength, e.g., the bonding was carried out at lower than optimal temperatures, and the crack would not deflect due to the increased value. In addition, the competition of the crack path in a well-bonded test vehicle is often observed as minute crack branching, as shown in Figure 15. These small-scale branched cracks often terminated within 1 μm. As the fracture progresses, the deflected path gradually gains favor.

**Figure 16:**
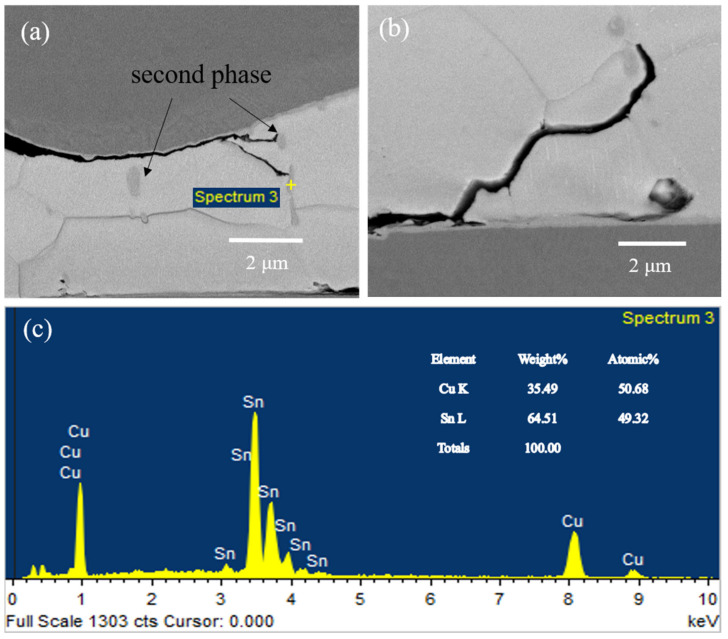
Influence of second-phase particles on crack propagation path: (**a**) crack tips meet the second phase and stop propagating; (**b**) The crack propagate around the second phase by deflection; (**c**) EDX analysis diagram.

The explanation for the crack branching is that the grain boundary is the low strength region and alternative crack propagation path. Here, a test vehicle with Cu–SnAg–Cu microbump structure was used to enhance the interfacial reaction, and the joint was ion-milled cross-sectionally before SEM observation to exhibit grain contrast, as shown in Figure 16. The second phase was identified as Cu_6_Sn_5_ IMC. IMC particles can be seen clearly in the junction of Sn grains, which is formed by Cu atoms diffusing along the grain boundary and precipitating in the junction in the form of Cu_6_Sn_5_ during the solidification process. These Cu_6_Sn_5_ particles play a significant role in the arresting and deflection of cracks. As can be seen in Figure 16a,b, crack tips meet the second phase and stop propagating. A higher driving force is required to either propagate around the second phase by deflection, or to continue through the second phase, the latter being less probable from an energetic point of view. Therefore, once arrested by the boundary junction, cracks would further proceed along the boundary of the IMC particle and Sn grain, while the preferred direction of all possible ones is related to the deflection angle, finally forming fracture patterns that differ from one sample to another in shape. The Ag_3_Sn IMC grains were believed to not have a significant impact on the crack propagation path since they were present in the form of a primary eutectic component located inside each Sn grain [17,18]. It has been previously reported that under thermal cycling or coupled thermomechanical–electrical load, the fatigue crack preferred an intergranular propagation path [19,20], in which case, the reconstructed grain structure and recrystallization might contribute to the weakening of grain boundary strength. This inclination seems to apply well to the highly dynamic and purely mechanical drop impact scenario. We can also reasonably suspect that if the interfacial IMC grows to a certain thickness that leaves visible voids due to the volume shrinkage effect, the bonding interface will be much weakened in that the crack will only propagate along the voided interface.

Combining the results in Figure 5, it can also be further deduced that the stage III of resistance change plays a significant role in determining the joint lifetime under drop impact, and one possible way to enhance the durability is to eliminate the grain boundaries; thus, the deflected path would cost higher energy than in a joint of the multi-grain solder layer. The research of controlling the grain number of the solder layer in a microbump joint is currently ongoing among various researchers [21,22].

## 6. Conclusions

In this paper, we report for the first time the cracking failure characteristics in microbump joints for chip-on-chip stacked interconnections. Experimental tests were carried out using a JEDEC standard test board to reveal the joint resistance change and the crack morphology. To elucidate the crack deflection during the joint degradation process, a local finite-element model was established to calculate the stress intensity factor at the crack tip, and the numerical results were further incorporated into a fracture mechanics model to obtain the crack deflection criteria. The main conclusions are summarized below:

(1) The main failure mode of microbump interconnections for 3D CoC packaging is that cracks were first initiated at the edge of the IMC–solder interface. After propagating along the interface for a distance, they deflected into the solder matrix, eventually penetrating the entire joint. The electrical resistance change is closely linked to the cracking progress.

(2) Stress intensity factor of a zero-thickness crack tip at the interface of the solder and IMC is calculated under quasi-static load by the method of interaction integral method. Both *K*_I_ and *K*_II_ increase with the increase in the crack length under the same load, and reducing the solder thickness causes higher SIF due to less alleviated mechanical mismatch.

(3) The crack propagation path is studied using a criterion based on energy release rate and fracture toughness. The calculation results show that the cracks on the interface between the solder and IMC will deflect into the solder matrix after extending to a certain depth along the interface. The deflection angle for crack initiation converges to 40° with the increase in crack length. The critical length of the main crack for crack deflection increases with the increase in solder thickness, which is experimentally confirmed by an actual drop test on samples with different solder heights.

(4) The crack propagation path in actual drop test samples was influenced by factors, including the actual strength of the bonding interface and the grain structure of the solder layer. Grain boundaries are the favored path for the deflected cracks.

## Figures and Tables

**Table 1:** Material properties of the main parts modeled as linearly elastic [14].

Part	Density (g/cm^3^)	Elastic Modulus (MPa)	Poisson Ratio
SAC305	0.00736	81,000	0.347
IMC	0.00855	114,000	0.318
Ni	0.0089	199,000	0.312

**Table 2:** Parameters of two-material SAC305–IMC interface.

**Parameters**	$E^{*}$	ϵ	β	$\mu_{1}$	$\nu_{1}$	$\mu_{2}$	$\nu_{2}$
Values	6.06 × 10^10^	−0.028	0.088	3.59 × 10^10^	0.314	1.388 × 10^10^	0.347

## Data Availability

Data sharing not applicable.
